# Concordance of the facial biotype between Bjork-Jarabak cephalometrics and photographic analysis of the facial opening angle

**DOI:** 10.4317/jced.60506

**Published:** 2023-06-01

**Authors:** Ana-Flavia Medina-Grandez, Luciana Llontop-Palma, Gustavo-Armando Ruíz-Mora, Yalil-Augusto Rodríguez-Cárdenas, Aron Aliaga-Del Castillo, Vinicius Dutra, Luis-Ernesto Arriola-Guillén

**Affiliations:** 1 School of Dentistry, Universidad Científica del Sur, Lima, Perú; 2 Division of Orthodontics, Faculty of Dentistry, Universidad Nacional de Colombia, Bogotá D.C, Colombia; and Associate Professor of the Division of Oral and Maxillofacial Radiology, School of Dentistry, Universidad Científica del Sur, Lima, Perú; 3 Division of Oral and Maxillofacial Radiology, School of Dentistry, Universidad Científica del Sur, Lima, Perú. Associate Professor of the Division of Oral and Maxillofacial Radiology, School of Dentistry, Universidad Nacional de Colombia, Bogotá D.C, Colombia; 4 Department of Orthodontics and Pediatric Dentistry, School of Dentistry. University of Michigan; 5 Department of Oral Pathology, Medicine, and Radiology, School of Dentistry, Indiana University; 6 Division of Orthodontics and Division of Oral and Maxillofacial Radiology, School of Dentistry, Universidad Científica del Sur, Lima, Perú

## Abstract

**Background:**

Analysis of the facial biotype can provide useful information for orthodontic diagnosis and can determine the type of growth of a patient to guide orthodontic treatment. The objective of this study was to determine the concordance of the facial biotype according to Bjork-Jarabak cephalometric analysis and photographic analysis of the angle of facial opening in Peruvian individuals.

**Material and Methods:**

This retrospective study included 244 cephalometric radiographs and frontal photographs of the same patients obtained from a database. The facial biotype (mesofacial, brachyfacial, or dolichofacial) was determined with the Björk-Jarabak polygon (cephalometric) and the angle of facial opening (photographic). Two trained investigators performed all the measurements. The concordance of the facial diagnosis was determined using correlations of the interclass coefficient and the kappa test. *p*<0.05

**Results:**

In cases with a mesofacial biotype, both analyses coincided in 60 individuals (68.2%), while in those diagnosed with a dolichofacial biotype, the analyses only coincided in 17 individuals (10.4%). There was no concordance between the two methods regarding the brachyfacial biotype since according to the angle of facial opening none of the individuals presented a brachyfacial biotype (kappa weighted test= 0.020, *p*=0.586).

**Conclusions:**

Cephalometric and photographic analyses should be complementary and one should not substitute the other. Attention should be focused on dolichofacial and brachyfacial biotypes, which showed less concordance between evaluations. So, more studies are needed to follow this line of research.

** Key words:**Facial biotype, cephalometry, photography, facial type, radiography.

## Introduction

Nowadays, determination of the facial biotype is fundamental for orthodontic treatment, since it guides the use of various orthodontic mechanics according to the patient’s growth pattern (1-4) and dentoalveolar compensation (5,6). The brachyfacial and dolichofacial biotypes present different variations in the dentoalveolar process that help on treatment planning, with the objective of planning specific mechanics considering patients´ growth pattern (6).

According to the literature, the facial biotype can be diagnosed by cephalometric analysis or other subjective clinical analysis methods based on soft tissues (7,8-10). Digital photographs also present various advantages and reliability over other clinical measurement methods, such as providing a permanent record, being easily reproducible, accurate, and having the possibility of performing multiple measurements (11-13). One of the most widely used clinical methods is the facial opening angle analysis due to its simplicity (14).

Cephalometry has shown to be reliable for the evaluation of skeletal and soft tissue structures and is considered the gold standard in the literature (15-17). The Björk-Jarabak polygon is a commonly used method for the determination of the facial biotype by cephalometric analysis (18,19). Agreement between cephalometric and clinical methods is the ideal condition for orthodontic diagnoses, although these results do not always coincide. It has been suggested that the disagreements found in the determination of facial types by cephalometric and photographic analysis are due to the fact that cephalometric studies evaluate bone tissues in profile views, while photographs evaluate soft tissues in frontal views (14). Moreover, some studies have evaluated the concordance between clinical and cephalometric diagnosis and reported different results (20-24). However, no study has used the Bjork-Jarabak polygon to compare concordance with the photographic method in the Latin American population. Therefore, this study aims to determine the concordance between Bjork-Jarabak cephalometric analysis and photographic analysis of the facial opening angle in the diagnosis of the facial biotype in Peruvian individuals.

## Material and Methods

This study was approved by the Institutional Research Ethics Committee of the Universidad Cientifica del Sur (CIEI-CIENTÍFICA) with registration code PRE-8-2022-00110 and was performed according to the Declaration of Helsinki (25).

This retrospective study was based on clinical records of 244 Peruvian individuals of both sexes attended in a private dental clinic that included frontal facial photographs and lateral head X-rays obtained at the same time.

The inclusion criteria for the study were complete information in clinical records, individuals with all teeth up to second molar and age between 13-45 years. The exclusion criteria were individuals with any known systemic disease affecting soft tissues, facial deformities, skeletal open bites, congenital anomalies, asymmetries or craniofacial trauma; having a history of orthodontic treatment or orthognathic surgery; facial hair masking landmarks or hindering identification and a history of any surgical/pharmaceutical therapy that may affect the facial structure or soft tissues.

Sample size calculations were determined using a sample size formula to estimate a proportion recording the following data: 95% confidence interval, power of the test 80%, expected proportion 80.02% (concordance between the diagnosis of the facial biotype in photographs and radiographs in mesofacial individuals obtained in a previous pilot study) and 5% accuracy. The minimum sample size required was 244 individuals(14-20). Sample size calculation was performed at: https://www.fisterra.com/formacion/metodologia-investigacion/determinacion-tamano-muestral/

The images of the lateral head radiographs were transferred to MicroDicom viewing software (version 0.8.1; Simeon Antonov Stoykov, Sofia, Bulgaria), without magnification, at a scale of 1:1 in order to perform the cephalometric measurements.

-Training and calibration 

Two researchers were trained to perform the measurements of the photographs and lateral cephalograms by a specialist with more than 10 years of experience, and the intra- and inter-examiner reliability were evaluated with Kappa test until obtaining values greater than 0.8 in all measurements.

-Cephalometric analysis measurement

**Figure 1:**
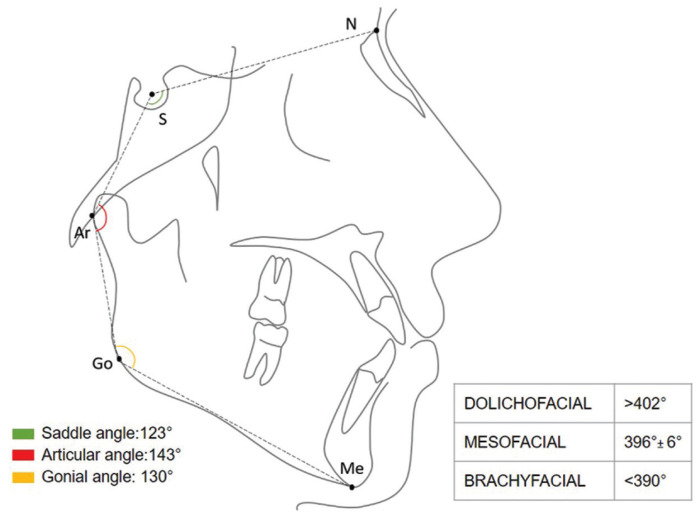
Determination of the facial biotype with the Bjork-Jarabak cephalometric method. and photographic analysis of the facial opening angle.

The cephalometric analysis was derived from the Björk and Jarabak polygon and included: NS-Ar (saddle angle), S-Ar-Go (articular angle), and Ar-Go-Me (gonial angle). Determination of the facial biotype was defined as the sum of these 3 angles that have an average value of 396° with a deviation of +/-6°. Therefore, according to the measurements, the facial biotype was: Mesofacial: 396° +/- 6°, brachyfacial: < 390°, and dolichofacial: > 402° (Fig. 1).

-Photo analysis measurement.

**Figure 2:**
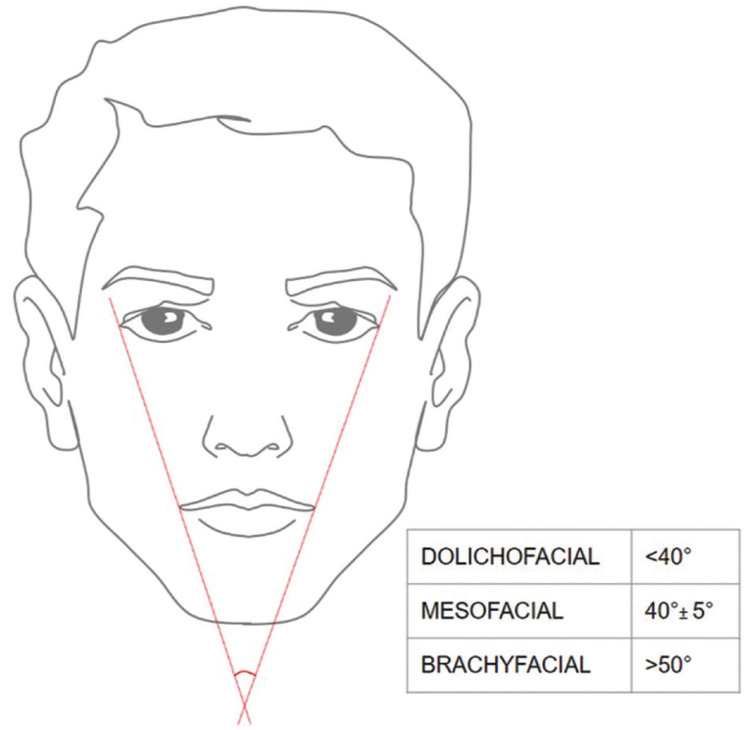
Determination of the facial biotype with the photographic analysis of the facial opening angle.

The photographic facial analysis was carried out by the determination of the angle of facial opening that is formed by two oblique lines (right and left) that pass from the external edges of the orbits to the labial commissure and then intersect and form an angle with an average value of 40° and a standard deviation of 5° as described by Viazis (26). Therefore, according to the measurements, the facial biotype will be: Mesofacial: 45° +/- 5°, brachyfacial: > 50° and dolichofacial: < 40° (Fig. 2). The photographs were measured in MicroDicom.

-Statistical analysis 

Statistical analysis was performed using the statistical program SPSS for Windows version 27.0.1. The means and standard deviation of the Bjork Jarabak cephalometric analysis and the photographic analysis of the facial opening angle were described. Subsequently, concordance of the diagnosis of the facial biotype with both methods was evaluated using the weighted Kappa test and the results were segmented according to gender. A *p* value < 0.05 was considered statistically significant.

## Results

The total number of cephalometries and frontal photographs analyzed was 244, of which 127 (22.52 ± 9.44 years old) belonged to females and 92 (20.55 ± 7.38 years old) to males (Table 1). The facial opening angle showed 88 mesofacial individuals (22.52 ± 8.97), and 156 dolichofacial individuals (20.55 ± 6.99). No brachyfacial individuals were found (Table 2). Björk-Jarabak analysis identified 61 mesofacial individuals (394.9 ± 3.36), 25 dolichofacial individuals (405 ± 2.82) and finally, 58 brachyfacial individuals (385.50 ± 3.50) were diagnosed (Table 3).

**Table 1:**
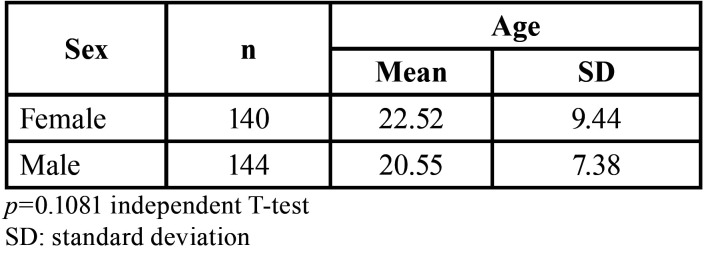
Initial characteristics of the sample evaluated by sex and age.

**Table 2:**
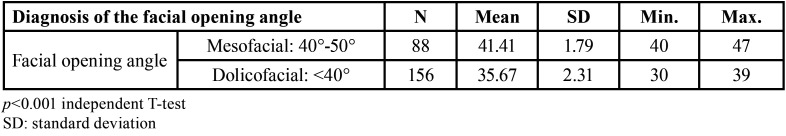
Evaluation of the angle of facial opening for the diagnosis of the facial biotype.

**Table 3:**
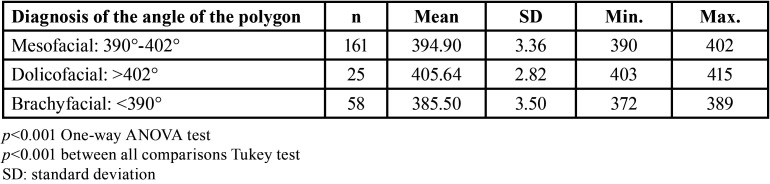
Evaluation of the Björk-Jarabak polygon for the diagnosis of the facial biotype.

Table 4 shows the low concordance of the diagnosis of the facial biotype between the angle of facial opening and the analysis of the Björk-Jarabak polygon. According to the results of the two methods, the values found showed no significant concordance (*p*=0.586) and did not exceed 70% in any of the comparisons. In cases diagnosed with a mesofacial biotype, the Björk-Jarabak polygon identified 161 individuals while the facial opening angle identified 88 individuals. The two methods coincided in the diagnosis of the facial biotype in only 60 individuals. On the other hand, in regard to the dolichofacial biotype, 25 were found using the Björk-Jarabak polygon and 156 with the facial opening angle method, coinciding only in 17 individuals. Finally, when evaluating the brachyfacial diagnosis, 58 individuals were diagnosed with the brachyfacial biotype using the Björk-Jarabak polygon, while none were identified with the facial opening angle method (Table 4).

**Table 4:**
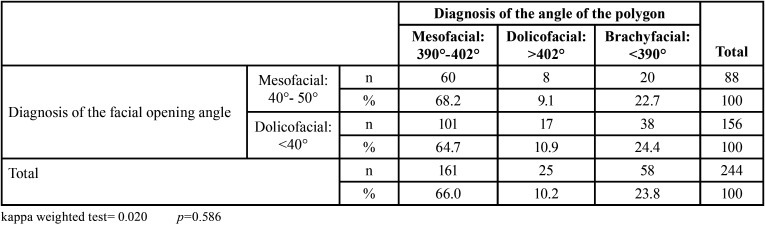
Concordance between the facial opening angle and the Björk-Jarabak polygon for diagnosing the facial biotype.

## Discussion

Diagnosis of the facial biotype is currently a key aspect for orthodontic treatment, since it guides the use of various mechanical orthodontics to create different responses when applied to individuals who have similar malocclusions but with different growth patterns. Thus, depending on the facial growth pattern and the dentoalveolar compensation that occurs, the brachyfacial and dolichofacial biotypes present different variations in the dentoalveolar process. The facial biotype must be in accordance with the facial aesthetics that are intended to be achieved with the treatment, and thus, accurate qualitative and quantitative analysis must be carried out to define the treatment, with the use of adequate auxiliary methods (7).

Herzberg (27) suggested that standardized photographs could be the best method for facial evaluation, since they allow detailed evaluation of measurements and proportions. In this sense, methods such as the facial opening angle have been used as an initial diagnosis to quantify the characteristics of the facial contour of individuals through photographs (29,30). Following this characterization, we can establish what auxiliary tests will be necessary to corroborate the diagnosis, such as the use of cephalometric analyses of the Bjork-Jarabak polygon (30,31). However, the amount of agreement between these two methods remains unknown and it is important to determine this correlation. For this reason, the aim of this research was to determine the concordance of the facial biotype according to Bjork-Jarabak cephalometric analysis and photographic analysis of the facial opening angle in Peruvian individuals.

For this study, we worked with a sample size of 244 participants, similar to previous studies, although there is no study to correlate these analyses. Some results in the literature have described a high level of agreement between cephalometric and photographic analyses (76.5%) (14), while other studies report little coincidence between the two analyses (k=-0.61) (20,21). These latter results coincide with those of our study, in which we found a coincidence of 68.2% in mesofacial, 10.4% in dolichofacial and 0% in brachyfacial biotypes, with a low weighted kappa value (0.020). From the clinical point of view the accuracy of one method is not superior to that of the other. Thus, it remains unclear as to which method an orthodontist should use to determine the facial biotype.

In our study, we observed that determination of the facial biotype by the facial opening angle was slightly more reliable compared to cephalometry (assessed by Bjork-Jarabak polygon) only in mesofacial individuals. Despite the fact that our study compared photography with cephalometry, clinical evaluation by expert orthodontists was not carried out, leading to the need for further investigation. However, taking into account the moderate agreement found, it is not yet possible to define which method would be the gold standard. Based on the above, we can conclude that in order to achieve an accurate diagnosis it is important to evaluate both cephalometric and photometric analyses. In addition, it is recommended that the two methods should be complementary and one should not substitute the other.

## Conclusions

Concordance between Bjork-Jarabak cephalometric analysis and photographic analysis of the angle of facial opening for determining the facial biotype in Peruvian individuals is low. These two analyses must be complementary and one should not substitute the other. Even when a sample size calculation was used, the results of this study cannot be generalized to the whole society, so more studies are needed to follow this line of research.
